# Supplementary material for: The Role of Tenascin-C on the Structural Plasticity of Perineuronal Nets and Synaptic Expression in the Hippocampus of Male Mice
Source: Biomolecules. 2024 Apr 22;14(4):508. doi: 10.3390/biom14040508 (PMC11047978; doi:10.3390/biom14040508)
Supplement: Supplementary file 1 [file biomolecules-14-00508-s001.zip › biomolecules-2931283-supplementary.pdf]

biomolecules

MDPI

Supplementary Material

The Role of Tenascin-C on the Structural Plasticity of Perineuronal Nets and Synaptic Expression in the Hippocampus of Male Mice

Ana Jakovljević [1], Vera Stamenković [2], Joko Poleksić [3], Mohammad I.K. Hamad [4], Gebhard Reiss [5], Igor Jakovcevski [5],* and Pavle R. Andjus [1],*

* Correspondence: pandjus@bio.bg.ac.rs (P.A.), igor.jakovcevski@uni-wh.de (I.J.)
Tel: +381-11-3032356 (P.A.), +49-2302-926-78663 (I.J.)

Supplementary Table S1. Acquisition settings used for confocal fluorescence imaging.

Experimental setup of confocal fluorescence microscopy								
	Objective	Laser Unit				Channel settings		
		Laser source	Wavelength	Power	Excitation transmission	Pinhole	Detector gain	Amplifier Offset
**WFA**	Plan-Neofluar 40x/1.30 Oil	Argon	488 nm	30 mW	35%	4 µm	672	-0.3
**PV**		HeNe1	543 nm	1 mW	30%		691	-0.3
**WFA**	Plan-Apochromat 63x/1.4 Oil	Argon	488 nm	30 mW	35%	2.2 µm	790	-0.3
**VGAT**		HeNe1	543 nm	1 mW	30%		763	-0.3
**VGLUT1**		HeNe2	633 nm	5 mW	30%		866	-0.3

Supplementary Table S2. Number of investigated PNNs and PV cells in the four subfields of the hippocampus

Group	Number of investigated PNNs	Number of investigated PV+ cells	Number of investigated PNNs for ultrastructure analysis	Number of investigated PNNs with VGAT and VGLUT1 puncta
		DG		
TnC +/+ SE	64	58	23	14
TnC +/+ EE	70	55	23	15
TnC -/- SE	77	49	23	17
TnC -/- EE	58	57	23	16
		CA1		
TnC +/+ SE	81	86	36	20
TnC +/+ EE	101	98	36	16
TnC -/- SE	90	68	36	17
TnC -/- EE	95	99	36	16
		CA2		
TnC +/+ SE	48	44	23	17
TnC +/+ EE	75	59	23	17
TnC -/- SE	90	65	23	14
TnC -/- EE	78	57	23	16
		CA3		
TnC +/+ SE	88	84	14	18
TnC +/+ EE	91	91	14	18
TnC -/- SE	101	104	14	17
TnC -/- EE	92	91	14	18

Supplementary Table S3. Results of statistical tests for every parameter in the four hippocampal subregions DG, CA1, CA2 and CA3.

Measured parameter	DG	CA1	CA2	CA3
	**Statistical test:<br>Two-way ANOVA, F value, degrees of freedom (df), p value**			
**PNN intensity**	Genotype<br>F (1,46) = 4.638<br>p = 0.037<br>No *post hoc* difference<br>Environment<br>F (1,46) = 2.177<br>p = 0.147<br>Genotype x Environment<br>F (1,46) = 2.071<br>p = 0.157	Genotype<br>F (1,47) = 1.017<br>p = 0.319<br>Environment<br>F (1,47) = 1.505<br>p = 0.226<br>Genotype x Environment<br>F (1,47) = 0.054<br>p = 0.817	Genotype<br>F (1,45) = 0.731<br>p = 0.397<br>Environment<br>F (1,45) = 0.501<br>p = 0.483<br>Genotype x Environment<br>F (1,45) = 0.334<br>p = 0.566	Genotype<br>F (1,45) = 0.367<br>p = 0.548<br>Environment<br>F (1,45) = 0.425<br>p = 0.518<br>Genotype x Environment<br>F (1,45) = 1.431<br>p = 0.238
**PNN number**	Genotype<br>F (1,45) = 0.031<br>p = 0.861<br>Environment<br>F (1,45) = 0.456<br>p = 0.503<br>Genotype x Environment<br>F (1,45) = 3.102<br>p = 0.085	Genotype<br>F (1,46) = 0.139<br>p = 0.711<br>Environment<br>F (1,46) = 0.459<br>p = 0.502<br>Genotype x Environment<br>F (1,46) = 0.291<br>p = 0.593	Genotype<br>F (1,45) = 8.694<br>p = 0.005<br>**Tukey *post hoc* test TnC+/+ SE vs. TnC-/-SE**<br>**p= 0.002**<br>Environment<br>F (1,45) = 0.022<br>p = 0.883<br>Genotype x Environment<br>F (1,45) = 6.688<br>p = 0.013<br>No *post hoc* difference	Genotype<br>F (1,45) = 0.021<br>p = 0.885<br>Environment<br>F (1,45) = 0.133<br>p = 0.717<br>Genotype x Environment<br>F (1,45) = 0.613<br>p = 0.438
**PNN+/PV+ intensity**	Genotype<br>F (1,42) = 5.482<br>p = 0.024<br>No *post hoc* difference<br>Environment<br>F (1,42) = 0.499<br>p = 0.484<br>Genotype x Environment<br>F (1,42) = 0.584<br>p = 0.449	Genotype<br>F (1,45) = 4.910<br>p = 0.032<br>No *post hoc* difference<br>Environment<br>F (145) = 0.301<br>p = 0.586<br>Genotype x Environment<br>F (1,45) = 0.022<br>p = 0.883	Genotype<br>F (1,43) = 0.728<br>p = 0.399<br>Environment<br>F (1,43) = 0.236<br>p = 0.630<br>Genotype x Environment<br>F (1,43) = 0.158<br>p = 0.693	Genotype<br>F (1,45) = 0.212<br>p = 0.648<br>Environment<br>F (1,45) = 0.538<br>p = 0.468<br>Genotype x Environment<br>F (1,45) = 0.601<br>p = 0.443
**PNN+/PV+ number**	Genotype<br>F (1,46) = 0.006<br>p = 0.939<br>Environment<br>F (1,46) = 0.315<br>p = 0.577<br>Genotype x Environment<br>F (1,46) = 0.594<br>p = 0.445	Genotype<br>F (1,46) = 0.011<br>p= 0.917<br>Environment<br>F (1,46) = 0.011<br>p = 0.917<br>Genotype x Environment<br>F (1,46) = 0.044<br>p = 0.834	Genotype<br>F (1,45) = 1.435<br>p = 0.238<br>Environment<br>F (1,45) = 0.000<br>p = 1.000<br>Genotype x Environment<br>F (1,45) = 0.283<br>p = 0.597	Genotype<br>F (1,45) = 0.113<br>p = 0.739<br>Environment<br>F (1,45) = 0.245<br>p = 0.623<br>Genotype x Environment<br>F (145) = 0.400<br>p = 0.531

**PNN+/PV-intensity**	Genotype<br>F (1,40) = 1.240<br>p = 0.273<br>Environment<br>F (1,40) = 6.408<br>p = 0.016<br>**Tukey *post hoc* test**<br>**TnC+/+ EE vs.**<br>**TnC+/+ SE**<br>**p=0.017**<br>Genotype x<br>Environment F (1,40)<br>= 3.737, p = 0.061	Genotype<br>F (1,44) = 0.008<br>p = 0.929<br>Environment<br>F (1,44) = 0.064<br>p = 0.902<br>Genotype x<br>Environment<br>F (1,44) = 0.160<br>p = 0.691	Genotype<br>F (1,41) = 0.640<br>p = 0.429<br>Environment<br>F (1,41) = 0.797<br>p = 0.377<br>Genotype x<br>Environment<br>F (1,41) = 0.003<br>p = 0.954	Genotype<br>F (1,42) = 1.830<br>p = 0.184<br>Environment<br>F (1,42) = 0.004<br>p = 0.953<br>Genotype x<br>Environment<br>F (1,42) = 1.449<br>p = 0.236
**PNN+/PV- number**	Genotype<br>F (1,46) = 0.243<br>p = 0.625<br>Environment<br>F (1,46) = 0.243<br>p = 0.625<br>Genotype x<br>Environment<br>F (1,46) = 1.727<br>p = 0.196	Genotype<br>F (1,47) = 0.249<br>p = 0.620<br>Environment<br>F (1,47) = 0.390<br>p = 0.536<br>Genotype x<br>Environment<br>F (1,47) = 0.249<br>p = 0.620	Genotype<br>F (1,45) = 9.612<br>p = 0.003<br>**Tukey *post hoc* test**<br>**TnC+/+ SE vs. TnC-/-SE**<br>**p< 0.001**<br>Environment<br>F (1,45) = 0.736<br>p = 0.396<br>Genotype x<br>Environment<br>F (1,45) = 11.356<br>p = 0.002<br>**Tukey *post hoc* test**<br>**TnC-/- EE vs. TnC-/-SE p= 0.023**	Genotype<br>F (1,45) = 0.285<br>p = 0.596<br>Environment<br>F (1,45) = 0.023<br>p = 0.879<br>Genotype x<br>Environment<br>F (1,45) = 0.023<br>p = 0.879
**PV+/PNN- number**	Genotype<br>F (1,46) = 0.591<br>p = 0.446<br>Environment<br>F (1,46) = 0.322<br>p = 0.573<br>Genotype x<br>Environment F (1,46)<br>= 2.092<br>p = 0.155	Genotype<br>F (1,47) =2.279<br>p = 0.138<br>Environment<br>F (1,47) = 2.693<br>p = 0.108<br>Genotype x<br>Environment<br>F (1,47) =2.279<br>p = 0.138	Genotype<br>F (1,45) = 0.035<br>p = 0.852<br>Environment<br>F (1,45) = 0.162<br>p = 0.689<br>Genotype x<br>Environment<br>F (1,45) = 0.950<br>p = 0.335	Genotype<br>F (1,45) = 0.285<br>p = 0.596<br>Environment<br>F (1,45) = 0.022<br>p = 0.882<br>Genotype x<br>Environment<br>F (1,45) = 0.384<br>p = 0.539
**PV+ number**	Genotype<br>F (1,46) = 0.021<br>p = 0.886<br>Environment<br>F (1,46) = 0.086<br>p = 0.771<br>Genotype x<br>Environment<br>F (1,46) = 0.339<br>p = 0.564	Genotype<br>F (1,46) = 0.488<br>p = 0.489<br>Environment<br>F (1,46) = 0.934<br>p = 0.339<br>Genotype x<br>Environment<br>F (1,46) = 2.090<br>p = 0.156	Genotype<br>F (1,46) = 0.758<br>p = 0.389<br>Environment<br>F (1,46) = 0.030<br>p = 0.863<br>Genotype x<br>Environment<br>F (1,46) = 1.216<br>p = 0.276	Genotype<br>F (1,46) = 0.758<br>p = 0.389<br>Environment<br>F (1,46) = 0.030<br>p = 0.863<br>Genotype x<br>Environment<br>F (1,46) = 1.216<br>p = 0.276
	**Statistical test:**<br>**Kruskal Wallis, H value, degrees of freedom (df), p value**			
**Nodes number in PNN mesh**	**H(3) = 15.761**<br>**p = 0.001**<br>**Dunn *post hoc* test**	H(3) = 1.637<br>p = 0.651	H(3) = 5.769<br>p = 0.123	H(3) = 0.826<br>p = 0.843

	**TnC+/+ SE vs. TnC-/- SE p = 0.001**<br>**TnC-/- SE vs. TnC-/- EE p = 0.026**			
**Nodes percentage in PNN mesh**	H(3) = 6.934<br>p = 0.074	H(3) = 4.373<br>p = 0.224	H(3) = 3.646<br>p = 0.302	H(3) = 4.668<br>p = 0.198
**Nodes connectivity degree**	H(3) = 4.862<br>p = 0.182	H(3) = 2.551<br>p = 0.466	H(3) = 4.918<br>p = 0.178	H(3) = 6.878<br>p = 0.076
**Average distance between nodes**	H(3) = 4.576<br>p = 0.206	H(3) = 4.410<br>p = 0.221	H(3) = 3.064<br>p = 0.382	H(3) = 4.497<br>p = 0.213
**Maximal distance between nodes - R95**	H(3) = 7.136<br>p = 0.068	H(3) = 4.322<br>p = 0.229	H(3) = 3.559<br>p = 0.313	H(3) = 2.013<br>p = 0.570
**VGAT density**	**H(3) = 9.436**<br>**p = 0.024**<br>**Dunn *post hoc* test**<br>**TnC+/+ SE vs. TnC-/-SE p = 0.014**<br>**TnC+/+ SE vs. TnC+/+EE**<br>**p = 0.037**	H(3) = 1.588<br>p = 0.662	**H(3) = 9.597**<br>**p = 0.022**<br>**Dunn *post hoc* test**<br>**TnC+/+ SE vs. TnC+/+EE**<br>**p = 0.004**<br>**TnC+/+ EE vs. TnC-/- EE p = 0.017**	**H(3) = 8.012**<br>**p = 0.046**<br>**Dunn *post hoc* test**<br>**TnC+/+SE vs.TnC+/+ EE**<br>**p = 0.016**<br>**TnC+/+EE vs. TnC-/- EE**<br>**p = 0.016**
**VGAT size**	H(3) = 5.183<br>p = 0.159	H(3) = 5.376<br>p = 0.146	H(3) = 5.521<br>p = 0.137	H(3) = 4.730<br>p = 0.193
**VGAT intensity**	H(3) = 0.218<br>p = 0.975	H(3) = 4.668<br>p = 0.198	**H(3) = 10.605**<br>**p = 0.014**<br>**Dunn *post hoc* test**<br>**TnC+/+ SE vs. TnC-/- SE**<br>**p = 0.015**<br>**TnC+/+ EE vs. TnC-/- EE**<br>**p = 0.044**	H(3) = 2.453<br>p = 0.484
**VGLUT1 density**	**H(3) = 7.569**<br>**p= 0.056**<br>**Dunn *post hoc* test**<br>**TnC-/- SE vs. TnC-/- EE**<br>**p = 0.011**	H(3) = 2.065<br>p = 0.559	H(3) = 3.423<br>p = 0.331	H(3) = 6.035<br>p = 0.110
**VGLUT1 size**	H(3) = 1.868<br>p = 0.600	H(3) = 2.039<br>p = 0.564	H(3) = 4.342<br>p = 0.227	H(3) = 2.658<br>p = 0.447
**VGLUT1 intensity**	**H(3) = 11.646**<br>**p= 0.009**<br>**Dunn *post hoc* test**<br>**TnC-/- SE vs. TnC-/- EE**<br>**p = 0.005**<br>**TnC+/+ SE vs. TnC-/- SE**<br>**p = 0.005**	H(3) = 0.103<br>p = 0.991	H(3) = 6.465<br>p = 0.091	H(3) = 2.056<br>p = 0.561

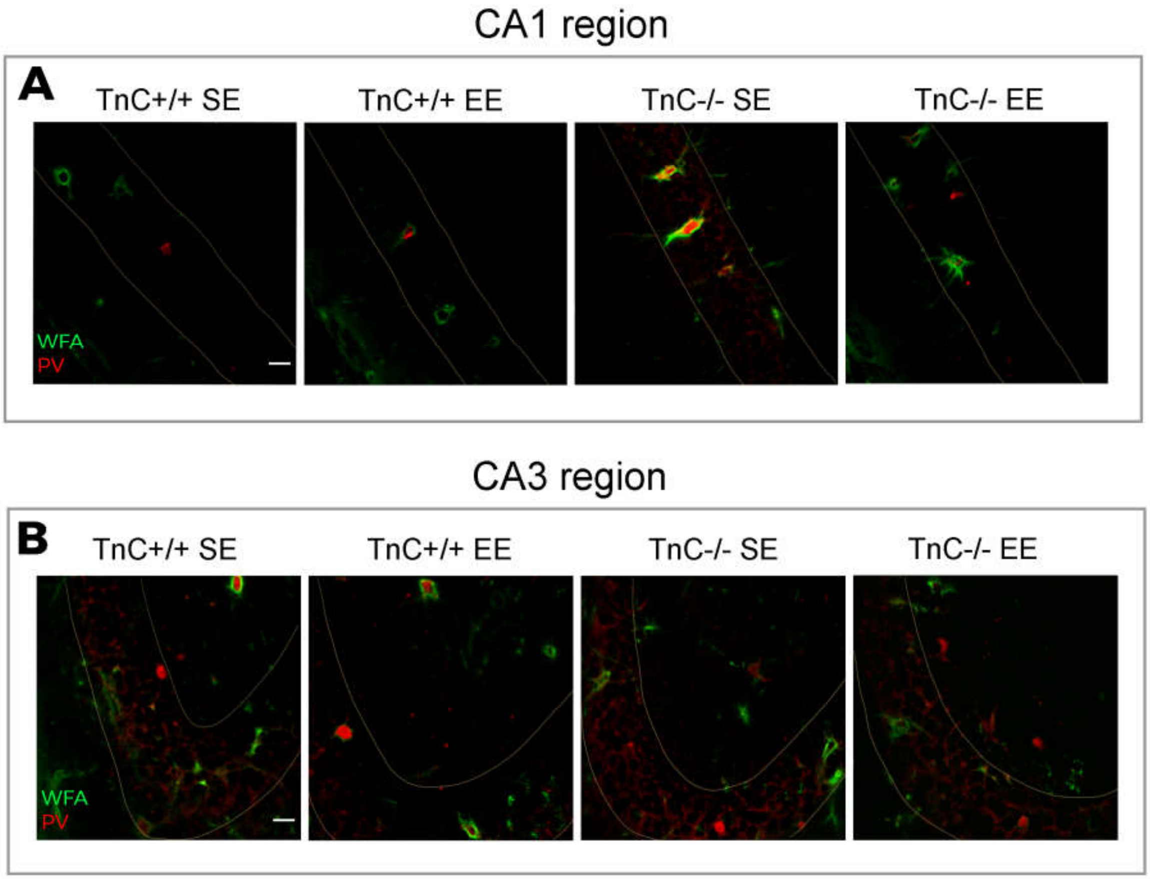

Supplementary Figure S1. Expression of PNNs and PV+ neurons in the region of CA1 and CA3 in TnC+/+ and TnC-/- mice after exposure to SE and EE. (**a, b**) Representative confocal images shows double immunofluoresent staining for PNN marker WFA (green). Pyramidal layer of CA regions is delineated with light yellow line. Scale bar (**a**): 50 μm.

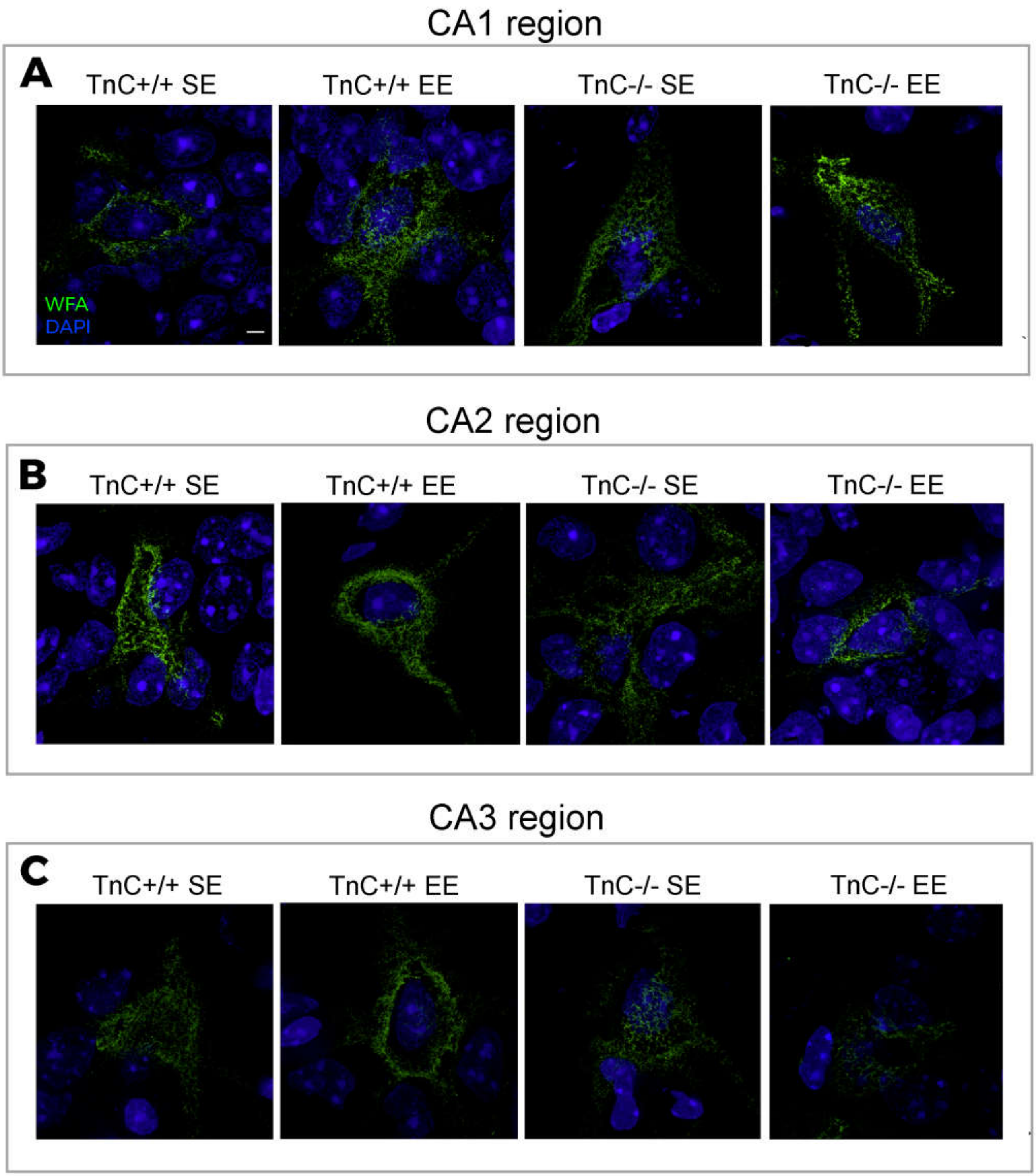

Supplementary Figure S2. Ultrastructure of PNN in the CA1 (**a**), CA2 (**b**) and CA3 (**c**) in the hippocampus of TnC+/+ and TnC-/- mice after exposure to SE and EE. Representative 3D super-resolution images from SIM shows double immunofluorescent staining for PNN marker WFA (green) and DAPI (blue). Scale bar (a): 3 µm.

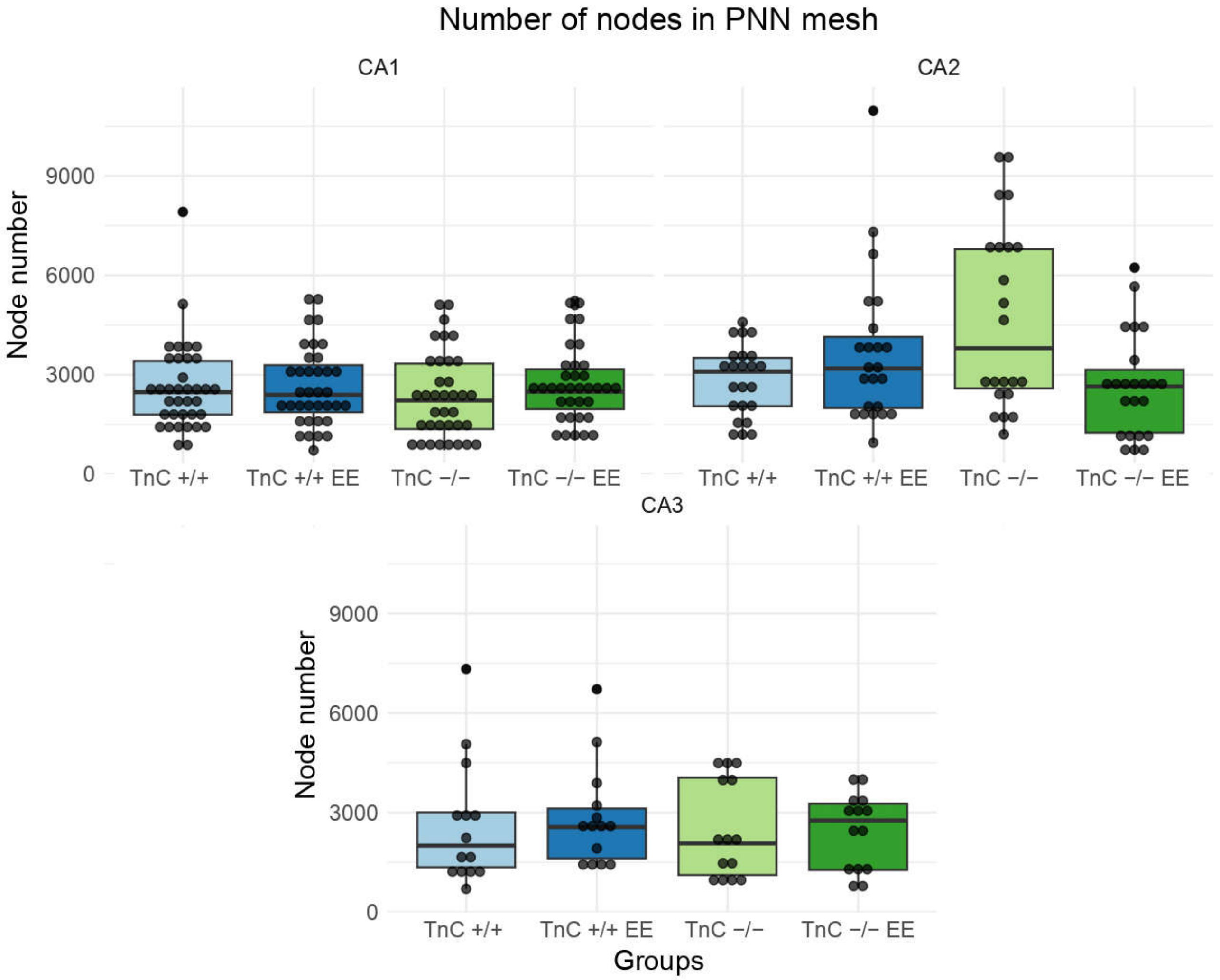

Supplementary Figure S3. Quantification of PNNs ultrastructure in the four subfields of the hippocampus of TnC+/+ and TnC-/- mice after exposure to SE and EE. Box plots represents median number of nodes in PNN mesh (4 animals/experimental group, 3 brain section/animal), whiskers extending from the box are indicating range of data within 1.5 times the interquartile range. No significant difference between groups is found.

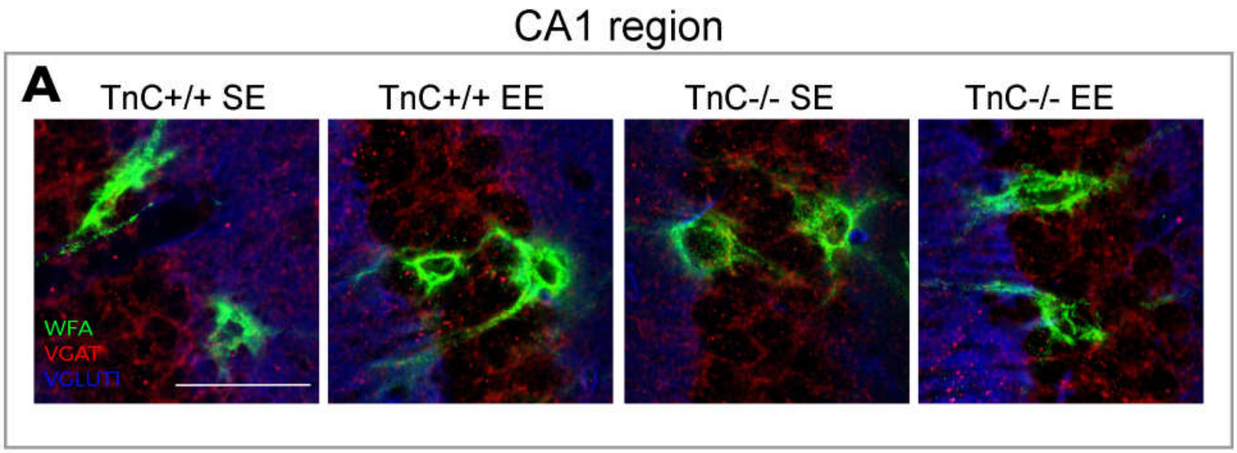

Supplementary Figure S4. Expression of inhibitory and excitatory synapses in the CA1 region in the hippocampus of TnC+/+ and TnC-/- mice after exposure to SE and EE. (**a**) Representative 3D confocal images from shows triple immunofluorescent staining for PNN marker WFA (green), inhibitory marker VGAT (red) and excitatory marker VGLUT1 (blue). Scale bar (**a**): 50 μm.

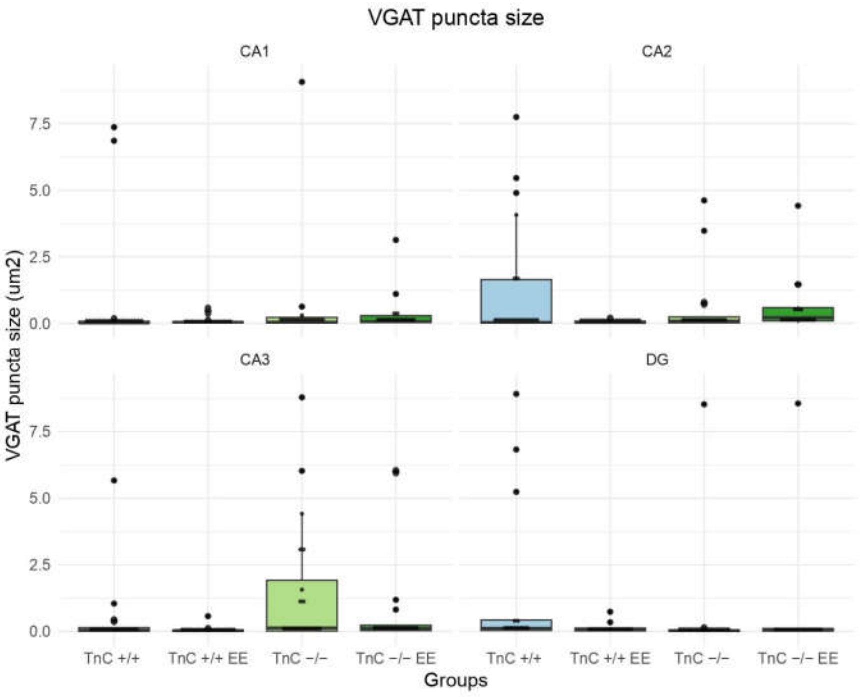

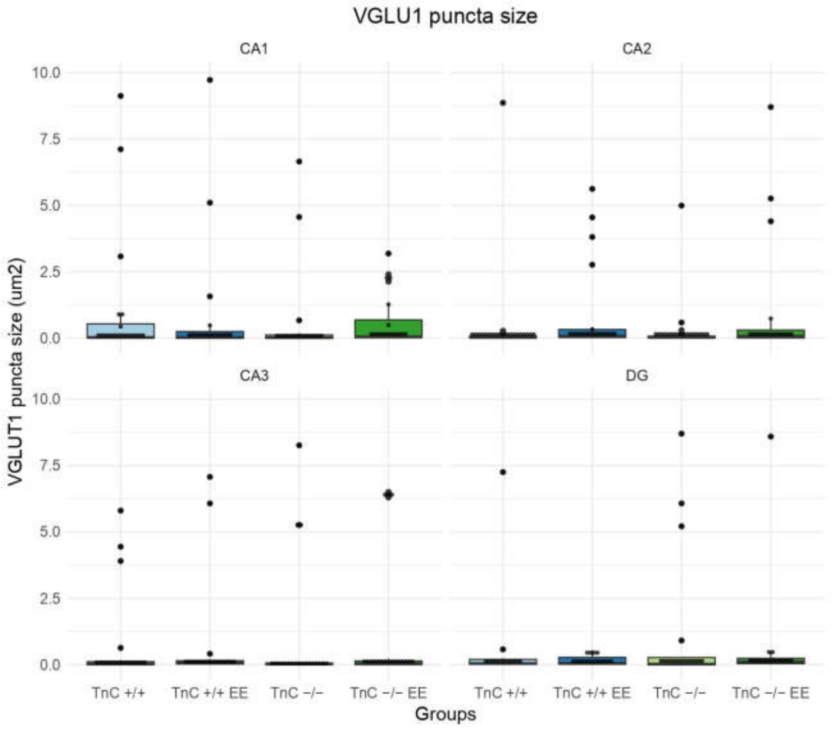

Supplementary Figure S5. Quantification of inhibitory and excitatory synapses size in the four subfields of the hippocampus of TnC+/+ and TnC-/- mice after exposure to SE and EE. Box plots represents size of inhibitory and excitatory puncta that are penetrating PNN (3-4 animals/experimental group, 3 brain section/animal), whiskers extending from the box are indicating range of data within 1.5 times the interquartile range. No significant difference between groups is found.
